# What Should We Aim for when Addressing Uncertainty from Serious Illness? A Stakeholder Focus Group Study

**DOI:** 10.1007/s11606-026-10364-z

**Published:** 2026-04-08

**Authors:** Rachel Chen, Joodi Mourhli, Ben Bowers, Jonathan Koffman, Stephen Barclay, Anna Spathis, Simon Etkind

**Affiliations:** 1 University of Cambridge, Department of Public Health and Primary Care (PHPC), Cambridge Biomedical Campus, Cambridge, UK; 2 Harvard Medical School, Boston, MA USA; 3 Hull York Medical School, Wolfson Palliative Care Research Centre, York, UK; 4 Cambridge University Hospitals NHS Foundation Trust, Cambridge, UK; 5 Cambridgeshire and Peterborough NHS Foundation Trust, Cambridge, UK

**Keywords:** uncertainty, serious illness, person-centred care, shared decision-making, relational care

## Abstract

**Background:**

Uncertainty is ubiquitous in serious illness. It defines experiences of diagnosis, current illness, and future prognosis and is experienced across physical, psychological, and temporal domains. Uncertainty impacts patients, carers, healthcare professionals and health systems; it cannot be eliminated, but it remains unclear how best to address it.

**Objective:**

We aimed to identify core goals and approaches when addressing irreducible uncertainty in serious illness to inform the development and implementation of effective interventions. We aimed to answer the question, “What should individual clinicians do to deliver ‘good care’ for patients experiencing irreducible uncertainty associated with serious illness?”

**Design:**

One-day rapid consensus workshop held in the United Kingdom, during which five mixed focus groups of 6–8 participants discussed uncertainty in serious illness and the goals of addressing it.

**Participants:**

Thirty-four participants, including 21 health and social care professionals, 8 researchers, 3 policymakers, and 3 patient representatives. Several participants held multiple roles. Age range 33–61 (median 47), 20 (67%) female.

**Approach:**

Focus group transcripts were analysed inductively using reflexive thematic analysis to generate a thematic model.

**Key Results:**

Analysis identified the overarching purpose of addressing uncertainty as “Finding security in uncertainty together,” comprising four key domains of: (1) relationships and trust across the care team; (2) personalisation of uncertainty management to an individual’s need and clinical context; (3) reframing uncertainty as normal and tolerable; and (4) moving forward together through shared decision-making and parallel planning.

**Conclusions:**

This study has generated a new model to approach uncertainty in serious illness. These findings suggest shifting the focus from the patient alone as an individual to strengthening relationality across the care team. Our findings emphasise the need to psychologically reframe living with uncertainty as a core goal, and to negotiate and align the needs of patients, carers, and clinicians when sharing uncertainty.

**Supplementary Information:**

The online version contains supplementary material available at 10.1007/s11606-026-10364-z.

## INTRODUCTION

Serious illness affects most people, whether they encounter it as a patient or carer. By Kelley and Bollens-Lund’s definition, “serious illness” is *“a health condition that carries a high risk of mortality AND either negatively impacts a person's daily function or quality of life, OR excessively strains their caregivers*.”^1^Examples include acute infection or injury requiring critical care, and life-limiting illnesses such as incurable cancer, organ failure, and dementia. Because definitions of “serious illness” generally have low sensitivity and high specificity^1^, there is no complete estimate of the population affected by it, but impacts are significant and widespread.

Uncertainty is integral to medicine^2,3^, and a core element of experience in serious illness, affecting patients, carers, and clinicians across all care settings^4,5^. Uncertainty is defined here based on Mishel and Han et al.’s conceptual work as “known unknowns,” characterized by inadequate understanding, a sense of incomplete, ambiguous or unreliable information, and subsequent difficulty in assigning meaning^6–8^.

While some uncertainties raised by serious illness can be reduced or eliminated (e.g. diagnostic uncertainty can often be resolved through testing)^7^, others are less easily resolved (e.g. prognostic uncertainty about the future course of illness and likelihood of recovery) and can only be anticipated in terms of likelihood and risk^7^. Such irreducible “aleatory” uncertainty forms part of experience in serious illness, and how it is interpreted therefore assumes significance^7^.

Uncertainty appraisal and subsequent management can have tremendous consequences. Uncertainty can be harmful and distressing, at times to the extent that people experience existential and psychological distress which threatens their sense of self^9,10^. For healthcare professionals (HCPs), uncertainty intolerance can lead to overuse of decision-making heuristics, excessive referrals, diagnostic testing and prescribing, and reduced shared decision-making^11–13^. Uncertainty intolerance, linked to challenges in identifying what a clinician doesn’t know individually and what cannot be known^2^, is also associated with increased practitioner distress and burnout^11,14–16^. However, Mishel established that uncertainty is not inevitably negative, but rather a neutral cognitive state that can be appraised as positive “opportunity” or negative “danger.”^8^ Her work reveals a window to adjust appraisal and psychologically adapt to irreducible uncertainty, which may strengthen relationships and generate new life perspectives for patients^17^. Furthermore, addressing uncertainty is an essential component of shared decision-making in serious illness contexts, thus supporting involvement in person-centred care (PCC)^18^.

Addressing irreducible uncertainty is critical, but what should we aim for when addressing it? Communication is key, but evidence regarding the effect of uncertainty communication is mixed, with evidence that over-communication about uncertainty decreases decision satisfaction^19^, while patients and carers report greater decision-making satisfaction when uncertainty is handled sensitively^20^. Furthermore, Brashers’ theory of communication and uncertainty management describes how individuals may aim to reduce, maintain, or increase uncertainty^21^. These findings indicate that there is no single best way to manage uncertainty: a person-centred approach, establishing individualised plans through thoughtful communication between patients, families, and care teams, is needed. However, such conversations commonly do not occur^22,23^.

Existing interventions such as the AMBER (Assessment, Management, Best Practice, Engagement, Recovery Uncertain) Care Bundle^24^, and the Psychosocial Assessment and Communication Evaluation (PACE)^25^, offer person-centred approaches to uncertainty by promoting clear communication and care planning. AMBER is tailored to unstable, hospitalized patients with uncertain recovery at risk of dying within 1–2 months, while PACE was designed for the intensive care unit (ICU) setting. These interventions require daily plan reassessment and frequent, structured conversations with patients and families about preferences should deterioration occur. While studies show these interventions are acceptable to staff and patients, some faced challenges operationalising eligibility criteria: for example, clinicians struggled to identify who was at risk of dying within 2 months during a trial of AMBER^26,27^. Furthermore, evidence of patient or clinician benefits is limited^28^.

It is difficult to assess the effectiveness of existing interventions to manage uncertainty when there is, as yet, no agreement on what the goals of addressing irreducible uncertainty in serious illness should be. Simply put, we do not know what “good” care looks like in these clinical situations. Using qualitative methods, this study asks: What should individual clinicians do to deliver “good care” for patients experiencing irreducible uncertainty associated with serious illness?

## METHODS

### Study Design

This qualitative focus group study was held during a one-day stakeholder workshop on serious illness uncertainty in the United Kingdom in 2023. We chose a stakeholder focus group approach based on the U.K. Medical Research Council^29^and Methods Of Researching End-of-life Care evaluation guidelines^30^, which recommend diverse stakeholder participation when conducting research into outcomes of care. Evidence also suggests that interdisciplinary and multi-perspective approaches can bridge the gap between patients’ and professionals’ understanding to establish a joint agenda^5^.

A group of patients and members of the public met during the study to support development of the study aims and methods and comment on the findings. This study was reviewed and approved by the Cambridge University Psychology Ethics Committee (Reference No: PRE.2022.125).

### Inclusion and Recruitment

Inclusion criteria for participants spanned multiple roles: 1) patient and carer representatives with lived experience of serious illness (see above definition); 2) health or social care professionals providing care to people with serious illness; 3) policymakers involved with commissioning or policymaking for this group; 4) academic researchers interested in serious illness uncertainty. Those under 18 years of age, and carers of patients aged under 18 were excluded. Clinicians and researchers were identified via local, regional and national clinical networks, focusing particularly on palliative care, geriatrics, intensive care, acute medicine, nursing, and general practice, and were recruited through email and online communications. People with lived experience were identified through local and regional patient and public involvement (PPI) fora.

### Data Collection

During a one-day workshop on serious illness uncertainty^31^, participants were informed that an optional part of the workshop involved participation in focus groups as part of this study. Before the focus groups, we obtained written informed consent and collected self-reported demographic information from participants.

Focus groups were mixed across professional disciplines and roles to elicit dialogue between different stakeholders and highlight relevant social dynamics^32^. The topic guide was developed by the research team and was informed by literature review: it covered the challenges of clinical uncertainty and desired outcomes of addressing uncertainty (Appendix A). Focus group recordings were transcribed verbatim and anonymised. Immediately afterwards, facilitators wrote 1–2 page reflections describing the discussions, conversation dynamics, and notable interactions.

### Analysis

We performed reflexive thematic analysis of anonymised transcripts^33^, with close attention to researcher positionality and reflexivity (see Text Box)^34^. Data analysis began with data familiarisation, in which one researcher (RC) read all transcripts with no previous knowledge of their contents, followed by inductive coding using NVivo 14 (Lumivero, Version 14, 2023). Another researcher (SE) double-coded one transcript with the aim of sense-checking and collaboration^34^. Codebooks were compared and found similar; differences were resolved through discussion and yielded new interpretations of the data. Subsequent rounds of coding incorporated these new interpretations and considered observations from facilitator reflections, self-reported demographic information, focus group composition, and participant roles and relationships.

**Table:** **Text Box.** Description of researcher positionality

**Paradigm** • This research question takes a social constructivist position which assumes that the nature of uncertainty is subjective, mediated by individuals' prior knowledge, expectations, and social interactions, rather than defined by a single objective truth^35^. These individual realities can be uncovered and characterised through qualitative methods such as the focus groups performed in this study^32^. Since the researcher's interpretation of the dataset is also affected by their subjective experiences and expectations, reflexivity is a critical component of the analysis^33^. **Subjectivity** • The researcher leading the analysis (RC) is a second-year U.S. medical student. As a student at the beginning of her medical training, she can code inductively with fewer prior expectations but may lack awareness of practical clinical limitations. Furthermore, she has primarily experienced the U.S. healthcare system and is less familiar with the National Health Service (NHS), where this dataset originated. Her international perspective may highlight interesting differences between countries but may also prevent her from noting U.K.-specific details. The researcher who double-coded one transcript (SE) is a U.K.-based palliative care consultant and assistant professor whose insights from clinical practice and academic theory also influenced the interpretation of the data. The researchers who collaborated on theme development (BB, JM) are clinical academic nurses who also contribute clinical and systems thinking insights to this analysis. The wider research team included 6 other researchers, many of whom hold clinical roles in palliative care or nursing. **Reflexivity** • Weekly self-check-ins were held to review newly developed codes and themes, guided by questions such as, "How have my position and personal experiences led me to this conclusion?"; thoughts were recorded in a reflexivity journal^36^. Researcher position and relationship to each theme were explicitly discussed before finalising any theme.	

Codes were condensed and organised, then developed into descriptive and analytic themes. A collaborative theme development meeting between four authors with different clinical and research backgrounds (RC, SE, BB, JM) deepened insights and helped refine the themes generated. Relationships between themes were visualised using handwritten and digital thematic maps and presented to the wider research team (described in Text Box). Relevant literature about medical uncertainty and person-centred care were incorporated at this stage^8,18,37–39^. Before finalising the themes, one researcher (RC) performed final sense-checking by returning to the data to confirm that themes accurately represented the data participants shared.

## RESULTS

We held five focus groups with 34 participants, 30 of whom provided demographic information. Age range was 33–61 (median 47). 20 (67%) were female. 24 participants identified as White (80%); three (10%) identified as Asian or Asian British; and three (10%) identified as Mixed or Multiple Ethnic Groups.

Many participants held multiple roles. Of the 30 participants who completed the survey, 21 participants were clinicians, of whom eight were also researchers. Clinicians’ roles included 12 palliative care specialists, three geriatricians, two nurses, one ICU specialist, one physiotherapist, one psychologist, and one general practitioner. Five participants were non-clinician researchers. Researchers represented fields including social science, anthropology, and medical education. Three participants were policymakers involved in commissioning for serious illness. Three participants were PPI representatives, one patient and two carers.

**Figure 1:**
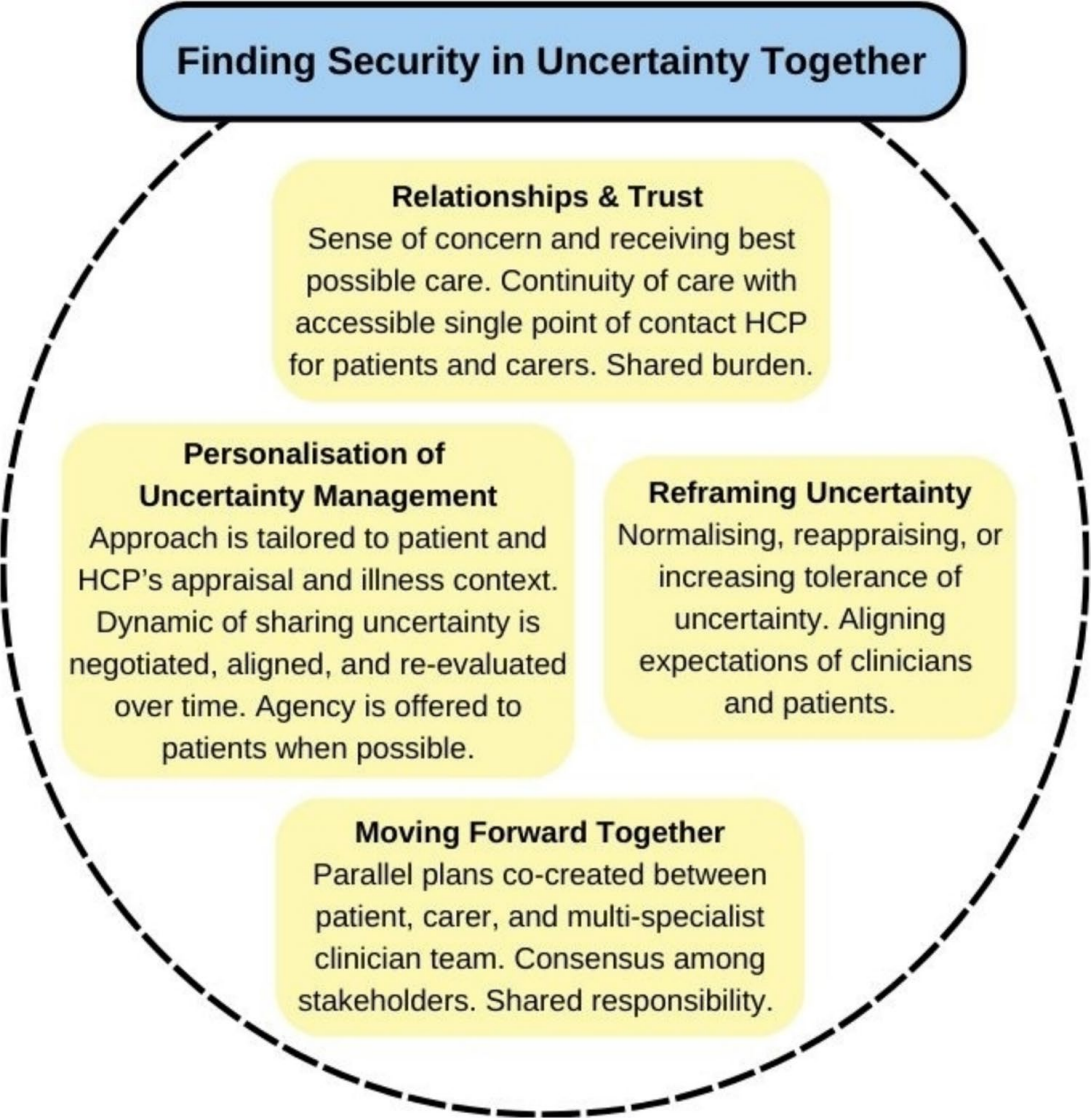
What should we aim for when addressing serious illness uncertainty? Thematic map of core purpose and supporting goals.

The core purpose of addressing uncertainty was identified as “Finding Security in Uncertainty Together.” All participants agreed that uncertainty was inevitable, and the ultimate objective should be feeling secure despite uncertainty by prioritising togetherness, both in the emotional experience of physician–patient-carer relationships and in the co-creation and communication of treatment plans. We generated four thematic goals to achieve this core purpose: (1) Developing “Relationships and Trust” between all involved in care; (2) “Personalisation of Uncertainty Management” to individual needs and clinical situation; (3) “Reframing Uncertainty” to be more normal, tolerable, or even positive; and (4) “Moving Forward Together” through shared decision-making and parallel planning for multiple possible scenarios. See Figure 1.

### Relationships and Trust

All focus groups emphasised “Relationships and Trust” as the foundation of finding security together when facing serious illness uncertainty. While one might expect that sharing uncertainty would undermine patients’ trust in their HCP’s abilities, participants discussed how concealing uncertainty could also damage the patient-provider relationship. The patient described her anger:

*“I just wish people would come clean and say they’re uncertain and that would have made me feel better, but they didn’t. And so they left me just not knowing for so long and my husband and I were having End-of-Life Care conversations because we just didn’t know how bad things were … that could have saved a lot of frustration and somewhat anger.”*

[Patient, Group 1]

Participants emphasised how in order to feel safe when HCPs share uncertainty with them, they need to trust the HCP. One carer felt that there were not always easy solutions in serious illness, so they primarily desired the security of having trusted, competent professionals by their side:

*“... it’s less important whether the healthcare professional gets it right or wrong… most patients will accept that not everything in life can be sorted out particularly when you are talking about palliative care… so it’s not uncertainty that’s more of a challenge, it is that trust and that feeling that the person who is looking after you know what they’re doing.”*

[Carer, Group 4]

Sharing the burdens of uncertainty brought comfort to both patients and HCPs. Many HCPs feared losing their professional credibility among colleagues if they shared their uncertainty, but they discussed how developing trust between colleagues and families can create a sense of security when facing uncertainty daily.

*“... developing a relationship and mutual trust and understanding, and that could be interprofessional but it could also be the relationship that the doctor has… with the patient and the family… or an organisational culture as well.”*

[ICU doctor, Group 1]

### Personalisation of Uncertainty Management

Participants widely considered “Personalisation of Uncertainty Management” to individuals’ needs and clinical situation to be important. While some people desire transparency, others do not want clinicians to communicate their uncertainty*.* HCPs also have varying degrees of willingness to reveal their uncertainty, often depending upon the expectations they set for themselves:

*“... what does your patient want to know? What do they need? But what do you need as well and where do you go with that if you can’t sort out what you need? Because you may do a really good job with your patient, you know, and be able to say ‘we haven’t got the answer to that,’ but that is a challenge to you as well, might not feel like you’ve done a good job…”*

[Nurse, Group 2]

Since patients and HCPs have varying personal preferences regarding sharing uncertainty, these preferences must be negotiated and aligned to reach an agreed approach. Successful bidirectional personalisation allows people to feel secure and valued within a close partnership that accommodates their individual needs. As needs change over time, re-evaluating and adjusting this fit deepens the sense of relational trust which is so critical to finding security in uncertainty together.

Although participants agreed that uncertainty could have distressing psychosocial impacts, they noted that its interpretation varied between individuals and that uncertainty was not uniformly a negative experience. For example, uncertainty could be an opportunity for hope for some patients, or an exciting intellectual challenge for HCPs.

*I love uncertainty, I don’t love anxiety, I love uncertainty, you know, I love that unpredictability of my clinical work in emergency work where you’re faced with these challenges and it suits my character and personality to work through them in a systematic way and get to the root of what’s going on, and it suits my character and my personhood to support someone through that journey which is, can be frightening, or overwhelming, but it brings out something in me which it may not in somebody else, but for me that uncertainty is something in my practice which really drives me.”*

[Emergency nurse and palliative care academic, Group 5]

Since individuals can view uncertainty differently and have varying preferences about sharing it, personalising uncertainty management approaches can help people feel secure within an accommodating team that understands and addresses their individual needs.

### Reframing Uncertainty

Participants recognised that one way to avoid distress and achieve a sense of security when facing uncertainty was to reframe it into a more acceptable form. This does not necessarily mean reframing uncertainty as a positive experience; doing so is not possible or desirable in many circumstances. Rather, normalising uncertainty may be more realistic. Since most serious illnesses involve elements of irresolvable uncertainty, many participants expressed that the ideal outcome of addressing uncertainty is for patients to better tolerate it.

*“... you can’t take away the uncertainty of ‘well when will I die’, there is certainty that the disease is progressing, but it’s that helping to sort of live with that uncertainty, to reduce how much the person is affected… ”*

[Nurse, Group 5]

Normalising uncertainty may be especially helpful for HCPs facing pressure to constantly present certainty. Many HCPs recalled being taught that there were correct solutions to all situations, so lacking certainty reflected personal shortcomings rather than clinical complexity.

*“… a lot of people internalise the uncertainty as a critique on their own practice, which is not necessarily right… [they] take that uncertainty as a failure of their own shortcoming, of their own work.”*

[Geriatrician, Group 5]

Normalising uncertainty may help manage expectations of both patients and HCPs, which can open opportunities for improved communication and collaborative working, so all individuals involved in care achieve a sense of relational closeness and security.

### Moving Forward Together

Participants emphasised that an important goal of addressing uncertainty was “Moving Forward Together” with a strong degree of consensus, care coordination, and co-creation of treatment plans. Patient and carer representatives discussed how poor coordination across multi-specialist teams caused unnecessary distress. The patient said:

*“I saw so many doctors and each one of them had a different opinion and when I wanted to see the specialists I was just told, you know, that they’ll be round at some point, see you in such and such and people just didn’t come. So in actual fact that makes you angry…”*

[Patient, Group 1]

The ideal approach participants identified was co-creating parallel plans to help everyone prepare emotionally and practically for a range of different possibilities. Doing so was perceived to offer a sense of control and safety, even creating some degree of certainty in uncertain situations:

*“... being able to have a frank conversation about… what the balance of options would be might actually create a sense of certainty for them… so if this happens we can do this, if this happens we can do that… ”*

[Palliative care doctor, Group 3]

In general, shared decision-making was felt to enable shared responsibility for outcomes. Participants described how relying on colleagues in a multidisciplinary team and including patients in decision-making can help lessen the burdens of responsibility:

*“... it’s sometimes easier to deal with the uncertainty if you all become responsible for the decision… that can help you deal with the sort of worries about the medicolegal things if you’re putting that decision out to share with your colleagues.”*

[Clinical academic, Group 1]

Co-creating parallel plans can help all stakeholders find security and togetherness with the certainty of having a plan and sharing decision-making responsibility with trusted partners.

Our analysis also identified three contextual influences that impact whether “Finding Security in Uncertainty Together” can be achieved: social attitudes and expectations, health system limitations, and HCP training. Each contextual influence affects the four key domains supporting this core purpose. Further information and detailed discussion are presented in Supplementary Information.

## DISCUSSION

### Summary of Findings

This study is the first to identify stakeholder-driven goals of interventions addressing irreducible uncertainty due to serious illness, building on the extensive prior literature of uncertainty in medicine to move towards measurable outcomes^2,8,21,37,40^. In short, theorists have described *what happens* in medical uncertainty, but this study begins to identify *what should happen* when addressing irreducible serious illness uncertainty. We developed the core purpose of “Finding Security in Uncertainty Together,” comprising four supporting key domains: “Relationships and Trust” between patients, carers, and all HCPs; “Personalisation of Uncertainty Management” to individuals’ needs and clinical situation; “Reframing Uncertainty” to be normalised so it can be better tolerated; and “Moving Forward Together,” through shared decision-making, parallel planning, and coordinated care.

### Contextualising Key Findings

1. Finding security together

Strengthening relationality was considered critical by most, especially when at the limits of professional expertise. This concept aligns with recent frameworks of shared decision-making and PCC^18^, while still allowing a more traditional approach for those who find greater relationality in those dynamics. The relationality emphasised in the core purpose of “Finding Security in Uncertainty Together” builds upon Mason’s model of (un)safe (un)certainty, in which clinicians and patients may feel “safe” or “unsafe” when facing uncertainty depending on how the dyad manages it^37^. “Safe uncertainty” requires people to feel comfortable with multiple potential outcomes, and to find paths forward that “fit” between professional and patient^37^. Our findings build upon this model by identifying “togetherness” as a critical component of engendering this safety. We chose “security” rather than “safety” for this reason; as McKenny critiques, “security” better captures the sense of confidence clients gain when they feel “in it together” with their provider, such that both clients and providers feel more prepared to face future risks^38^. This “togetherness” creates a sense of certainty through trusting, relational care and cohesive teamwork. Existing literature supports the importance of “security” in serious illness^39^. Here, we extend the core need for security and relationality to interprofessional care team dynamics and broader situations of uncertainty.

In practice, finding security “together” requires relational, whole-team rather than individual-focused care. This requires moving beyond the mainstream PCC approach of eliciting a patient’s choices and executing them to “address the relational, and often subtly negotiated, nature of care” between patients and caregivers^41^. Delivering quality care in uncertain situations requires finding security “together,” by negotiating agreements around handling uncertainty between all members of the care team. This concept is incorporated in existing interventions like the AMBER care bundle^24^. However, our findings suggest it may be helpful to place greater emphasis on parallel planning for multiple future possibilities, including optimistic and pessimistic scenarios^42^. Imagining and preparing for multiple potential outcomes alongside trusted providers can strengthen the sense of relationality which drives “Finding Security in Uncertainty Together.”

2. Reframing uncertainty

Despite calls to change medicine’s approach to uncertainty^43^, most existing interventions do not aim to shift individuals’ appraisal of uncertainty or ability to cope with it, rather focusing on communication and care coordination^44^. The existential impact of prognostic uncertainty is unavoidable in many serious illnesses, so models of “good” person-centred care should include some aspect of meaning-making. Mishel discussed the possibility of adaptation to chronic uncertainty by developing a “probabilistic” way of thinking in which uncertainty is “accepted as the natural rhythm to life.”^17^ Doing so would allow individuals to accept various contingencies, which aligns with our finding that parallel planning is especially critical in serious illness uncertainty. This study uses stakeholder data to confirm that for people experiencing irreducible serious illness uncertainty, psychological reframing is an essential pillar of “good care.” By including “Reframing Uncertainty” as a key domain, this model specifically acknowledges the psychological challenges that people managing serious illness face. It opens a space to perceive uncertainty as an opportunity for improving care processes and outcomes rather than as solely a threat in some contexts.

Few evidence-based interventions exist to facilitate reframing uncertainty. Current approaches such as AMBER and the Serious Illness Conversation Guide (SICG) implicitly normalise uncertainty by discussing possible decline early and frequently, but do not overtly seek to reframe it^24,45^. Brashers discusses encouraging people to”reappraise uncertainty… to change their perspective to view uncertainty as a normal part of life.”^21^However, neither these interventions nor Brashers’ theory explicitly guide patients, carers, or HCPs through the process of reconceptualizing or normalizing uncertainty psychologically. Acceptance and commitment therapy and meaning-centred psychotherapy are interventions which may fill this role, through empowering patients to adapt to negative experiences by focusing on value-based goals, staying in the present, and supporting uncertainty tolerance^46,47^. However, such interventions are often lengthy. Developing briefer tools that can be integrated into routine clinical care would help professionals offer personalised options for “Reframing Uncertainty.”

Before uncertainty can be reframed, it must be recognised and acknowledged. Interventions such as the Recognize, Acknowledge, Partner, and Seek Support framework^48^ and the uncertain recovery communication guide^49^may be useful to improve acknowledgement of uncertainty, but require evaluation. Improved HCP training, a contextual influence identified in this analysis, is also key to improving how uncertainty is addressed. Medical trainees are taught to project confidence and control over uncertainty^3,50^. Simpkin & Schwartzstein argue that because clinical practice deals in ambiguous idiosyncrasies rather than correct answers, medical education must be reframed around managing uncertainty going forward^43^. Scenario-based learning, reflective supervision, or uncertainty tolerance exercises may help normalise uncertainty and equip professionals to deliver relational care more confidently amidst uncertainty.

### Clinical and Research Implications

Given the potentially severe impacts of uncertainty on HCPs, patients and health systems, there is an urgent need to address and mitigate the effects of irreducible uncertainty in serious illness^5,11^. Current interventions such as AMBER are promising and acceptable to patients and staff, but it remains unclear whether they are effective^26,28^. Various interventions aimed at uncertainty management or communication, including PACE and SICG, contain elements of “good care” identified in this analysis, but there is limited evidence of success due to lack of an assessment rubric^25,44,45,51^. This study has taken a step towards evaluation by developing the outline of an evaluative model, but the goals identified here are not yet quantifiable outcome measures which can be used to assess effectiveness of care. Further work is needed to identify, adapt, or develop metrics which operationalise the core purpose of “Finding Security in Uncertainty Together” and the four thematic goals supporting it while remaining brief, comprehensive, and sensitive to change^29^.

The key domains presented in this study offer insight into effective care in situations of irreducible uncertainty in serious illness. They could inform interventions’ mechanisms of action and can guide optimal clinical practice, specifically supporting existing empirical interventions which achieve “security” and “togetherness.” For clinicians, educators, and policymakers, this model could be used to inform reflective tools**, **team debrief structures**,** or communication resources to help address uncertainty in everyday practice and train HCPs to manage uncertainty. Within some patient-carer-HCP teams, uncertainty could be used as an opportunity to improve team-based, relational care.

### Strengths & Weaknesses

We recruited participants with extensive expertise and experience with uncertainty, which increased the chance of a rich dataset and of successfully identifying outcomes. This was a rigorous qualitative study, using interdisciplinary focus groups to facilitate collaborative discussion. Several steps were taken to enhance the trustworthiness of this analysis, including double-coding, incorporating facilitator reflections, maintaining a reflexivity journal, collaborating with the wider research team, and sense-checking findings with relevant theory and literature. Support from a patient and public involvement group helped to ensure relevance to patient priorities.

Most study participants were HCPs, with only one patient and two carers participating, so these findings mainly represent clinicians’ views. Although patient and carer views largely aligned with those of HCPs, and the views expressed by patients and carers in other similar research^6^, sample size was low and alignment may have been influenced by perceived power dynamics within the group. Further study should explore patient and carer perspectives separately from clinician influence. All participants were already interested in the subject so findings may not be transferable to people who are indifferent about uncertainty. Furthermore, this study was U.K.-focused and included mainly White British participants. Care should be taken when thinking about how these findings may apply in different sociocultural contexts. While participants highlighted the importance of normaliing uncertainty, they did not discuss processes by which to achieve it. Future studies should aim to identify uncertainty normalisation and reframing processes.

## CONCLUSION

In exploring the goals of addressing irreducible uncertainty associated with serious illness, this study identified the core purpose of “Finding Security in Uncertainty Together.” Our findings highlight the importance of relational trust, personalised care, reframing uncertainty, and shared decision-making. By centring togetherness, our findings build upon existing theories of uncertainty and emphasise the relational and team-based nature of achieving security in care. We extend current frameworks by advocating for reframing uncertainty not only as a challenge, but also as a potential opportunity for psychological adaptation and meaning-making.

By identifying key domains of good care in uncertain contexts, this study lays the foundation for evaluating uncertainty interventions, which will enable the improvement of existing interventions and the development of new ones. Ultimately, creating a rubric of goals for addressing serious illness uncertainty will help establish effective, evidence-based uncertainty management strategies that can be delivered to more patients experiencing it. Future work should develop and test brief, scalable tools to support HCPs, patients, and carers in navigating uncertainty.

## Supplementary Information

Below is the link to the electronic supplementary material.

Supplementary file1 (DOCX 19 KB)

Supplementary file2 (DOCX 24 KB)

Supplementary file3 (DOCX 23.0 KB)

## Data Availability

The data generated and analysed during the current study are not publicly available due to privacy concerns but are available from the corresponding author on reasonable request.

## Declarations

### Additional Notes

RC conducted this research at University of Cambridge, Department of Public Health and Primary Care (U.K.) but has since moved to Harvard Medical School (U.S.A.).
