# Supplementary material for: What Should We Aim for when Addressing Uncertainty from Serious Illness? A Stakeholder Focus Group Study
Source: J Gen Intern Med. 2026 Apr 8;41(10):2725–33. doi: 10.1007/s11606-026-10364-z (PMC13421509; doi:10.1007/s11606-026-10364-z)
Supplement: Supplementary file 1 — Supplementary file1 (DOCX 19 KB) [file 11606_2026_10364_MOESM1_ESM.docx]

**COREQ Checklist**

1. Which author/s conducted the interview or focus group?
   1. Dr. Simon Etkind conducted the focus groups.
2. What were the researcher's credentials? *E.g. PhD, MD*
   1. PhD, MBBS.
3. What was their occupation at the time of the study?
   1. Associate Professor, Palliative Care Consultant.
4. Was the researcher male or female?
   1. Male.
5. What experience or training did the researcher have?
   1. PhD with dissertation focused on qualitative research of serious illness uncertainty; years of clinical experience as a palliative care consultant treating patients with serious illness.
6. Was a relationship established prior to study commencement?
   1. Yes.
7. What did the participants know about the researcher? e*.g. personal goals, reasons for doing the research*
   1. Participants first participated in an educational module about managing uncertainty in serious illness, so they were aware of the goals and reasons for doing the research.
8. What characteristics were reported about the interviewer/facilitator? e.g. *Bias, assumptions, reasons and interests in the research topic*
   1. See Figure 1 for description of subjectivity.
9. What methodological orientation was stated to underpin the study? *e.g. grounded theory, discourse analysis, ethnography, phenomenology, content analysis*
   1. See Figure 1 for description of methodological orientation.
10. How were participants selected? *e.g. purposive, convenience, consecutive, snowball*
    1. Purposive.
11. How were participants approached? e*.g. face-to-face, telephone, mail, email*
    1. Telephone, email.
12. How many participants were in the study?
    1. 34.
13. How many people refused to participate or dropped out? Reasons?
    1. 0.
14. Where was the data collected? e*.g. home, clinic, workplace*
    1. Workplace conference room.
15. Was anyone else present besides the participants and researchers?
    1. No.
16. What are the important characteristics of the sample? e.g. *demographic data, date*
    1. See Results section for full description of participants.
17. Were questions, prompts, guides provided by the authors? Was it pilot tested?
    1. Yes. See Appendix A for topic guide for focus groups.
18. Were repeat interviews carried out? If yes, how many?
    1. No.
19. Did the research use audio or visual recording to collect the data?
    1. Yes – audio recording.
20. Were field notes made during and/or after the interview or focus group?
    1. Yes – facilitator reflections were analysed alongside focus group transcripts.
21. What was the duration of the interviews or focus group?
    1. Approximately 1 hour each.
22. Was data saturation discussed?
    1. No.
23. Were transcripts returned to participants for comment and/or correction?
    1. No.
24. How many data coders coded the data?
    1. 1-2. See methods for description of double-coding process.
25. Did authors provide a description of the coding tree?
    1. Yes – see methods for description of inductive coding process.
26. Were themes identified in advance or derived from the data?
    1. Derived from the data.
27. What software, if applicable, was used to manage the data?
    1. NVivo 14 (Lumivero, Version 14, 2023).
28. Did participants provide feedback on the findings?
    1. No.
29. Were participant quotations presented to illustrate the themes / findings? Was each quotation identified? e*.g. participant number*
    1. Yes. Quotations were identified by group number and occupation.
30. Was there consistency between the data presented and the findings?
    1. Yes.
31. Were major themes clearly presented in the findings?
    1. Yes.
32. Is there a description of diverse cases or discussion of minor themes?
    1. Yes.
