# Supplementary material for: What Should We Aim for when Addressing Uncertainty from Serious Illness? A Stakeholder Focus Group Study
Source: J Gen Intern Med. 2026 Apr 8;41(10):2725–33. doi: 10.1007/s11606-026-10364-z (PMC13421509; doi:10.1007/s11606-026-10364-z)
Supplement: Supplementary file 2 — Supplementary file2 (DOCX 24 KB) [file 11606_2026_10364_MOESM2_ESM.docx]

**Supplementary Information**

Three contextual influences shape whether the four key domains of “Finding Security in Uncertainty Together” can be achieved: social attitudes and expectations, health system limitations, and HCP training. Each contextual influence impacts all the key domains identified in this analysis.

Social attitudes such as public ideas of the health system and HCPs’ ideas of themselves set the expectations surrounding uncertainty and who should manage it. For example, some patients still expect HCPs to have all the answers with complete certainty, while others are open to HCPs expressing uncertainty. Differences in social expectations sometimes align with different demographic groups:

*“F3: …you’re still on a pedestal up here, you are, you know, and in some respects they think you’re God and they think you know everything and I speak to a lot of elderly people that are like that.*

*M3: But do you think that’s a generational change? Because I think you’re right because the patients that are more elderly that come to us, there is that, but I do find that the younger generation and those within certain cultural groups, the expectations are very different.”*

 [Patient (F3) and ICU doctor (M3), Group 1]

These expectations originate within as well as outside the healthcare profession. One clinical psychologist expressed her hypothesis that perhaps HCPs’ high expectations for the quality of care they expect themselves to deliver adds to the distress they feel when faced with uncertainty.

*“... in terms of the burnout in staff because I wonder whether some of it comes from patients or whether some of it’s coming from staff putting pressure on themselves to take on more responsibility or to perfectly follow somebody up in the community or whatever it might be and kind of understanding those expectations both to inform healthcare but also whether there is some discussions early on that need to happen about … realistic expectations… ”*

[Psychologist, Group 3]

The effect of these social expectations is heightened by health system limitations such as sufficient resources, time, and the medicolegal environment. One doctor’s comment highlights the exacerbating interaction between social expectations and systemic limitations:

*“... One of the most stressful things is not being able to promise that we will be able to send out community teams or that we will be able to prescribe x or that we’ll be able to do this when it’s necessary… there’s an expectation that ‘the state will care for me, I paid for my taxes, when I’m old they’ll care for me’ and that’s no longer true or only partly true. And I think that makes the, adds huge uncertainties because there isn’t a safety net anymore.”*

[Palliative care doctor, Group 3]

In general, the availability of resources impacts whether delivering personalised care is feasible. Moreover, trusting relationships are only possible when HCPs have adequate time to spend with the patient and their family. However, as one community geriatrician laments, most HCPs operate under intense time pressure daily:

*“... the pressure on actual clinical workload, finding time to have these conversations, which aren’t just necessarily with the individual, with the extended family, when you’re actually we need the next patient in the bed, it’s really, really difficult…”*

[Geriatrician, Group 5]

Furthermore, for HCPs to feel safe practising shared decision-making, shared responsibility must be protected in the medicolegal environment. Several HCPs discussed how fear of legal retaliation drives their defensive approach to uncertain clinical situations. Without systemic protections and facilitators, HCPs may be reluctant to change their behaviours.

*“... how I make decisions is always a bit of a defensive approach…  if I’m standing… in front of the Courts…  Can I justify what I’m doing? … How protective or not is the GMC or legal system in recognising uncertainty?... there’s a lot of psychological distress amongst doctors who are under say GMC investigations, you know, there’s case reports of doctors committing suicide for example … Are doctors making more of a decision which is based on a defensive approach from the medicolegal system which may not support them as opposed to what’s in the patient’s best interests?”*

[Palliative care doctor, Group 1]

These health system limitations are especially powerful contextual influences because they also impact HCP burnout and resilience. Burnout poses a significant barrier to building trusting relationships and personalised care, while building resilience would facilitate achieving each of the identified key domains.

Finally, HCP training shapes different specialists’ attitudes toward uncertainty and presents an opportunity for HCPs to learn to share uncertainty productively. Contrasting cultures of addressing or ignoring uncertainty in different specialist fields lead to inconsistent care and prevent multispecialist teams from reaching the consensus, cohesion, and trust that patients desire in their care teams. An ICU consultant highlights the deficits in his own field:

*“So palliative care there’s going to be more, there’s more communication skills training, there’s more getting to know your patient, whereas intensive care’s more about… risk scoring systems… I think they’re kind of trying to sort of push uncertainty to one side and say ‘actually we’re going to try and make things as certain as possible to make decisions’ whereas palliative care is the opposite, palliative care is again about knowing not just the patient, you know, their psychosocial background, spiritual needs etc, etc, which as intensivists we’re probably guilty of not really looking too much into I would think.*”

[ICU doctor, Group 1]

In all focus groups, participants agreed that HCPs were not trained to handle the uncertainty which awaited them in clinical practice. Participants also agreed that improving HCP training to handle uncertainty would greatly facilitate achieving the key domains identified in this analysis.

*“... my memory of medical school was that, FY1, FY2, was that it was very strongly hospital focused… then when I did an FY2 job in general practice I found it completely overwhelming, and in retrospect that was being completely unfamiliar with that style of uncertainty and the watchful waiting thing did not come natural at all, I was completely unprepared for it. So I think there is something there about medical training at the moment not really preparing people for that kind of uncertainty.”*

[Palliative care doctor, Group 5]

Contextual influences such as HCP training, social attitudes and expectations, and health system limitations greatly affect the feasibility of achieving key domains of “good care” addressing irreducible uncertainty. While they may appear to be solely barriers to successful individual care team interactions, they may also represent potential levers for change in large-scale interventions to address uncertainty.
