# Supplementary material for: What Should We Aim for when Addressing Uncertainty from Serious Illness? A Stakeholder Focus Group Study
Source: J Gen Intern Med. 2026 Apr 8;41(10):2725–33. doi: 10.1007/s11606-026-10364-z (PMC13421509; doi:10.1007/s11606-026-10364-z)
Supplement: Supplementary file 3 — Supplementary file3 (DOCX 23.0 KB) [file 11606_2026_10364_MOESM3_ESM.docx]

# Appendices

### **Appendix A:** Focus group topic guide

**Topic guide for focus groups**

**Instructions for facilitator**

- The focus group is one hour in length.
- **Please go through the ‘introduction’** ***at the start of the session***, which will take approximately 5 minutes. ***All of the ground rules must be highlighted, especially confidentiality and the need to maintain patient/colleague anonymity.***
- It is essential to cover all topics in the session, in particular the generation of a list of research priorities. We have suggested timings for each topic but please feel free to use your discretion.
- It is not essential to cover every prompt under each topic - these are there to help direct and focus the discussion, but you are free to use your discretion.
- **Try to let the conversation flow between group members**, rather than interrupting with prompts and probes. Only use prompts if it is necessary to refocus the discussion.
- **After the focus group, please write a short (1-2 paragraph) reflection about how you felt it went as soon as possible afterwards.** It would be helpful to have your comments on group dynamics or the tone/mood of the session, as well as how you felt as the facilitator.
- **Each facilitator will have 2 voice recorders- please use both.** **We’d advise that you press ‘record’ just before the ‘introduction’, so you don’t forget to do so.** Please hand over the recorders after the session: all recordings will be securely downloaded at the end of the day. So long as this is successful, the voice recorder can then be wiped clean.

**Facilitator’s introduction (five minutes)**

1. **The facilitator introduces self**
2. **The facilitator checks that all have completed a consent form and demographics questionnaire, remind the participants about audio recording for research purposes and reassures them about confidentiality and anonymity.**
3. **The facilitator briefly explains the purpose of the discussion:** “We are all here because we’re interested in the uncertainties that can occur in serious illness, and how this can affect people with illness and those caring for them. We will all have different views and perspectives on uncertainty and the aim of today’s discussion is to share experiences and build a consensus on the priorities for future research. This focus group is the first step of that consensus process”
4. **The facilitator explains the ground rules for the focus group discussion:**

- “We want you to do the talking, one person at a time
- We would ideally like to hear from everyone, but you are free to participate as much or as little as you like
- There are no right or wrong answers: please be sensitive to others’ views and experiences.
- What is shared in this room stays in this room: please keep everything you hear confidential
- When discussing examples from your practice, please be mindful of the anonymity of your patients and colleagues”

**Focus group discussion (approx. 55 minutes)**

**Experiences ( 10 minutes) Ask participants to introduce themselves in turn, and give a short example of how they have experienced a situation of uncertainty concerning serious illness**

**The effects of uncertainty: What effects can uncertainty have in situations of serious illness? (10 minutes)**

- Consider physical, practical, psychological, existential, social, and temporal domains.
- Consider effects at individual and system levels.

**When confronted with uncertainty, what should we be aiming for? What outcomes are important? (10 minutes)**

- Individual and system-level outcomes
- Outcomes for patients, carers, health professionals

**What are the key research questions and priorities to achieve these outcomes? (15 minutes)**

- What situations is it important to investigate uncertainty in?
- What aspects of uncertainty do we need to know more about?
- What aspects of addressing or managing uncertainty are important?

**Summary and list writing: Facilitator (or scribe) writes a list of the items identified in section 4 & asks if there are other areas or items to add to the list. (10 minutes)**

Thank participants and reiterate confidentiality.

**Item list: (facilitator to list all research questions and priorities identified during the focus group discussion)**

………………………………………………………………….………………….…………………..…………………………………………………………………………………

………………………………………………………………….………………………….…………………………………………………………………..………………………….

…………………………………………………………………..…………………………. …………………………………………………………………..………………………….

…………………………………………………………………..…………………………. …………………………………………………………………..………………………….

…………………………………………………………………..…………………………. …………………………………………………………………..………………………….

…………………………………………………………………..…………………………. …………………………………………………………………..………………………….

………………………………………………………………….………………………….. …………………………………………………………………..………………………….

…………………………………………………………………..…………………………. …………………………………………………………………..………………………….

…………………………………………………………………..…………………………. …………………………………………………………………..………………………….

…………………………………………………………………..………………………….

**Facilitator notes:**Please write a short (1-2 paragraph) reflection below about how you felt the session went as soon as possible afterwards and return it to Simon: [sde23@medschl.cam.ac.uk](mailto:sde23@medschl.cam.ac.uk). You may wish to comment on group dynamics or the tone/mood of the session, as well as how you felt as the facilitator.

**Facilitator reflection:**

**Facilitator name:**

**Group number:**
